# Impact of COVID-19 on subnational variations in life expectancy and life disparity at birth in India: evidence from NFHS and SRS data

**DOI:** 10.1186/s13690-023-01170-8

**Published:** 2023-09-04

**Authors:** Pawan Kumar Yadav, Suryakant Yadav

**Affiliations:** 1 Department of Bio-Statistics and Epidemiology, International Institute for Population Sciences (IIPS), Mumbai, 400088 India; 2 Department of Community Medicine, Sikkim Manipal Institute of Medical Sciences, Sikkim Manipal University, Gangtok, Sikkim 737102 India

**Keywords:** Subnational mortality, Age pattern, Age at death, Covid-19, Life expectancy, Life disparity

## Abstract

**Background:**

Measuring life expectancy and life disparity can assist in comprehending how the COVID-19 pandemic has affected the mortality estimates in the Indian population. The present study aims to study the life expectancy and life disparity at birth at the national and subnational levels before and during the COVID-19 pandemic using the NFHS and SRS data.

**Methods:**

The measures Life expectancy at birth (${e}_{0}$) and Life disparity at birth (${e}_{0}^{\dagger}$) were computed for the non-pandemic and pandemic years from NFHS (2015–16), SRS (2015) and NFHS (2019–21), SRS (2020) respectively at the national and Subnational level in India. Using NFHS data for the 36 states and SRS data for the 22 states, the study calculates ${e}_{0}$ and ${e}_{0}^{\dagger}$ by total, male and female population.

**Results:**

The ${e}_{0}$ for male and female decline from 64.3 years and 69.2 years in 2015–16 to 62.9 years and 68.9 years in 2019–21. The ${e}_{0}$ shows a drop of approximately 1.4 years for males and 0.3 years for females in the pandemic year 2019–21 when compared to the non-pandemic year 2015–16. At the subnational level ${e}_{0}$ shows a decline for 22 states in person, 23 states in males and 21 states in females in the pandemic year 2019–21 as compared to the non-pandemic years 2015–16. The ${e}_{0}^{\dagger}$ shows a increase for 21 states in person, 24 states in females and 17 states in males in the pandemic year than non-pandemic year. The findings shows a significant losses in ${e}_{0}$ and gains in ${e}_{0}^{\dagger}$ for males than females in the pandemic year as compared to the non-pandemic year at the subnational level in India.

**Conclusions:**

COVID-19 pandemic has decreased ${e}_{0}$ and increased ${e}_{0}^{\dagger}$ in the pandemic year 2019–21 at the national and subnational level in India. COVID-19 had a significant impact on the age pattern of mortality for many states and male, female population and delayed the mortality transition in India.

**Supplementary Information:**

The online version contains supplementary material available at 10.1186/s13690-023-01170-8.

**Table:** 

**Text box 1. Contributions to the literature**
• This is the first study to calculate the life expectancy and life disparity estimates for all 36 states of India and compare the estimates during the pandemic year (2019–21) and non-pandemic year (2015–16)
• Out of 36 states analyzed, the COVID-19 pandemic led to losses in 22 states life expectancy in person with large losses of life expectancy in 23 states for males and 21 states among females
• Losses in life expectancy were largely attributable to increased mortality in adult and older age groups in the pandemic year 2019–21 compared to the non-pandemic year 2015–16
• These findings contribute to recognized gaps in the literature and observed that COVID-19 has generated a huge mortality toll at the national and sub-national level in India, with a disproportionate number of deaths occurring among the male population

## Background

Life expectancy at birth ($${e}_{0})$$ in India has increased through the twentieth century and the first decade of the twenty-first century [1–3]. Men and women born in 1970–75 could expect to live on average 50.5 and 49 years, respectively. By 2015–19 $${e}_{0}$$ had risen by 22.1 years for women ($${e}_{0}$$=71.1) and 17.9 years for men ($${e}_{0}$$=68.4), respectively [4]. This unexceptionable increase in $${e}_{0}$$ is derived from a substantial decrease in mortality rates from infectious diseases, population-wide public health actions (e.g., water sanitation, vaccination campaigns, the introduction of antibiotics), and increasingly extensive improvements in the socio-economic well-being of the Indian population. These factors significantly decreased infant, child, young age, and old age mortality in India [5, 6].

In India, ${e}_{0}$ has increased significantly in recent decades, while inequality in life expectancy at birth (${G}_{0}$) and disparity in life expectancy at birth (${e}_{0}^{\dagger}$) have decreased [7–9]. National, state, and yearly changes in Indian population mortality characteristics have been widely studied for a long time. To better understand the mortality conditions in India, several researchers have looked at age-specific percent contributions to ${\mathrm{e}}_{0}$ and sex differentials in ${\mathrm{e}}_{0}$ and ${\mathrm{G}}_{0}$ [10–19]. The recent Covid-19 pandemic has affected many countries in different ways, and the works of literature have emphasized that the impact of the Covid-19 pandemic is unevenly distributed in population groups and geographical areas [20–25]. A recent study by Yadav et al. (2021) has shown that India's ${e}_{0}$ dropped by 2.0 years in the pandemic year 2020 versus the non-pandemic year 2019. The 35–79 age group explains the drop in ${e}_{0}$ [19]. Further, another study aiming to track the losses in ${e}_{0}$ globally, found that Indians lost 2.6 years in their ${e}_{0}$ due to the Covid-19 pandemic in the year 2021 [26].

Increases in life expectancy in most modern cultures over the past few decades have prompted discussions regarding whether and to what degree people value potential increases in their own lifespans. In most modern societies, the motivation for long life and life extension is a new and developing problem. Little is known about what inspires people of various ages to want to live long lives under the various conditions that old age may entail [27]. Life expectancy can also throw more light on the overall impact of a crisis like COVID-19 on population health and allow comparisons with prior population health circumstances because it is sensitive to the ages at which deaths occur and because it is consistent through time [28–30]. Life disparity, which has been more frequently documented in population health research, is a supplementary indicator of population health with implications for public health planning. While life expectancy is used as an indicator of average mortality, life disparity emphasises on the variation in lifespans within a population as an additional measure of mortality.

Due to differences in the distribution of age at deaths, two populations may have the same life expectancy (i.e., average) but varying levels of life disparity. Thus, life disparity offers a complementary viewpoint that represents how increases in population health are distributed unevenly throughout a population, which has significant ramifications for organizing social and medical services [31]. Life expectancy inequality has tended to decline throughout the course of the twentieth century, according to trends from Indian states. This is because life expectancy and the modal age at death have both grown [9, 31, 32]. However, the age patterns underlying improvement in each measure vary. Lower mortality across all ages leads to longer life expectancy. The number of lives saved at the youngest ages has a direct correlation with the strength of the relationship between life expectancy and life disparity: the more lives saved at the youngest ages, the stronger the relationship is. However, more lives need to be saved at younger ages than older ones, typically below life expectancy, for life disparity to decrease when life expectancy is growing. By compressing the distribution of deaths, this raises the similarity of death ages. Life expectancy and life disparity at birth assess the cumulative impact of the pandemic on population health. Since both measurements are based on a single year, they might not accurately reflect the life cycle of a cohort. However, life expectancy is a helpful indicator of typical lifespans, and life disparity sheds light on the ambiguity surrounding the age at death [33]. We demonstrate how the pattern of mortality improvements across time and age may be used to illustrate patterns of change in life expectancy and life disparity. This paper provides a thorough evaluation of the mortality effects of the COVID-19 in the pandemic years (2019–21) and compares with non-pandemic years (2015–16) by taking into account two measures: life expectancy, and life disparity at national and subnational level in India.

In India, the Sample registration system (SRS) is the only source that provides the age-specific death rates and life table estimates annually for India and its 22 bigger states with a population of 10 million and above [4]. India consists of 28 states and 8 union territories; in sum, there are 36 states. To date, not a single study has provided estimates of ${e}_{0}$ for all 36 states in India. The present study which uses two rounds of NFHS data for 2015–16, 2019–21 and two rounds of SRS data for 2015 and 2020 respectively, will fill this research gap and provide ${e}_{0}$ estimates for all 36 states in India and compare the ${\mathrm{e}}_{0}$ estimates from both the data sources NFHS and SRS. Further, this study will estimate and compare the ${e}_{0}^{\dagger}$ for India and all 36 states for 2015–16 and 2019–21 from NFHS and 22 states for 2015 and 2020 from SRS, respectively. This study has also examined the changes in ${e}_{0}$ and ${e}_{0}^{\dagger}$ estimates in India and 36 states from 2015–16 to 2019–21, respectively. The age-specific mortality rates are obtained at India and state levels, and ${e}_{0}$ and ${e}_{0}^{\dagger}$ are estimated for the total, male and female population. In particular, this study will provide the evidence of mortality differentials between men and women at national and sub-national (state) levels for 2015–16 and 2019–21 from NFHS and 2015 and 2020 from SRS in India.

### Theoretical background: life expectancy at birth $({e}_{0})$ and life disparity at birth $({e}_{0}^{\dagger})$  

${e}_{0}$ is an important summary indicator of a population's health, mortality, and well-being. ${e}_{0}$ outlines the mortality trends in all age categories, including children, adolescents, adults, and the elderly. ${e}_{0}$ reflects the nation's socioeconomic conditions and the quality of healthcare infrastructure [34–36]. ${e}_{x}$ is an indicator of average mortality; it hides considerable variations in lifespans, which can be obtained by an index of lifespan variation or inequality, e.g., lifespan inequality by Gini coefficient, life disparity, standard deviation, interquartile range, and Years of life lost.

${e}_{0}^{\dagger}$ is a well-known mortality measure, and it measures the uncertainty in the timing of deaths at the individual level and divergence in the underlying population health at the macro level [31, 37]. The disparity in lifespan is an essential demographic measure because uncertainty about how long a person will live can have significant implications for the decisions over the life course, such as the age of retirement, savings, and investments in education [38]. Therefore, the first statistical moment of the distribution of lifespans, known as ${e}_{x}$, and the second statistical moment must be ${e}_{0}^{\dagger}$ are used to measure the actual cost of Covid-19 disease on the longevity of men and women in India and states. Monitoring Inequality in life expectancy or life disparity can reveal crucial information about disparities in mortality and health. Life expectancy disparity have been linked with large part of socioeconomic status. In order to reduce life expectancy inequality and ensure that rising life expectancy benefits all people, regardless of their age or other socioeconomic characteristics, countries should raise the life expectancy for all socioeconomic groups to match the rate of the highest socioeconomic group.

While ${e}_{x}$ and ${e}_{0}^{\dagger}$ had a strong negative correlation historically [33, 39, 40], some recent researchers have found a positive association between these two indicators in some countries and subpopulation groups showing higher mortality in younger ages [41–43]. Therefore, defining the health status of a population based on ${e}_{x}$ alone can lead to ignoring significant disparities in health equity in general. For example, two populations with the same life expectancy can have very different levels and trajectories of life disparity.

Mortality reduction at any age increases ${e}_{x}$ and vice versa, while it impacts the variation in lifespan differently. When ${e}_{x}$ increases, then to decrease ${e}_{x}^{\dagger}$, more lives need to be saved at younger than older ages. What is "younger" or "older" depends on a clearly defined threshold age that distinguishes between early and late death, typically at or near the level of ${e}_{x}$.While a reduction in mortality in old age will increase variance, a drop in mortality below the threshold age will compress mortality and hence decrease variation [44].

There are very few studies investigating into variability in mortality schedules at the subnational level in India. However, this study addresses the knowledge gap by estimating life expectancy and life disparity before covid-19 pandemic from NFHS (2015–16), SRS (2015) and during the pandemic from NFHS (2019–21) and SRS (2020). This paper examines a change in life table estimates of ${e}_{0}$ and ${e}_{0}^{\dagger}$ in the non-pandemic year 2015–16 and pandemic year 2019–21. The main objective of the study is to examine how the COVID-19 disease affects the life expectancy at birth and life disparity at birth at the national and subnational level in India.

## Methodology

### Data

In this study, we examined the changes in mortality patterns in India and its 36 states by sex, residence, and time using two recent National Family Health Survey data: NFHS (2015–16) and NFHS (2019–21). We have also used SRS data for 2015 and 2020 for India and 22 states to compare the estimates of ${e}_{0}$ and $({e}_{0}^{\dagger})$ from NFHS. This study used the data on 601,599 and 636,699 households available in NFHS (2015–16) and NFHS (2019–21), respectively [45, 46].

NFHS collects information about the deaths of any usual member of the households who died in the past three years prior to the date of the interview, i.e., January 2013 until December 2016 for the NFHS (2015–16) [45] and January 2016 to April 2021 for NFHS (2019–21) [46]. The NFHS provides the population by age, sex, and residence, as well as 74,945 and 81,340 deaths during 2015–16 and 2019–21, respectively. NFHS sample weights are used to compute deaths and populations by age groups. Mortality rates are assumed to remain constant during the survey, given that age-specific mortality rates for 2015–16 and 2019–21 are divided by three to calculate the annual age-specific mortality rates. Therefore, mortality rates are obtained for the population characteristics such as total, men, and women population in India. NFHS-5 fieldwork for India was conducted in two phases: Phase-I from 17 June 2019 to 30 January 2020, covering 17 states and 5 UTs, and Phase-II from 2 January 2020 to 30 April 2021, covering 11 states and 3 UTs — by 17 Field Agencies and gathered information from 636,699 households, 724,115 women, and 101,839 men. We analyzed all 36 states covered in the two phases and did not separate the results phase-wise from NFHS-5. The death registration of NFHS-5 has observed Seventy-one percent of deaths of usual household members were registered with the civil authorities, constituting 83% of urban households and 66% of rural households. The age-specific registered deaths observed 51% of deaths at age 0-4, 76% at age 25-34, and 75% at age 35 and above. NFHS-5 has collected 81,340 deaths in total in the two phases; Phase-I has collected 36,410 deaths from the 17 states and 5 UTs, and Phase-II has collected 44,930 deaths from the 11 states and 3 UTs. Phase-I has covered 28,708 deaths that occurred in the year 2016-18 and 7,656 deaths that occurred in the years 2019-21. Phase II has covered 21,750 deaths that occurred in the years 2016-18 and 23,152 deaths occurred in the years 2019-21. NFHS is the only data source that can be used to calculate the age-specific mortality rates for all 36 states. NFHS has limitations, as we can only estimate the single years ASDR based on the deaths of any usual household member in the last three years preceding the survey. Therefore, we can only compare the mortality estimates during the pandemic years (2019-21) and non-pandemic years (2015-16) based on the Single years ASDR. NFHS-5 (2019-21) has collected the deaths till April 2021, whereas The First case of the COVID-19 pandemic was reported on 30 January 2020 in India. Thus, NFHS-5 had also collected the Covid deaths occured in the pandemic years 2020-21 [46]. The SRS uses a dual record approach to gather information on vital statistics from representative sampling villages and urban blocks in India. The baseline survey captures information about the usual sample population resident. An enumerator regularly records every birth and death that occurs in the sample region. After six months, an independent supervisor updates the vital events of households. To find cases that are not matched, the data from the two sources is compared. To increase the accuracy of data, the unmatched cases are validated in field.

### Methods

#### Annual mortality rates calculation and life table construction

This study calculated the age-specific mortality rates by sex in India, and its 36 states for 2015–16 and 2019–21, respectively. We used SRS Age-specific death rates for 2015 and 2020 to construct life tables for India and 22 states. There were 466 missing deaths in 2015–16 and 682 missing deaths in 2019–21; these missing deaths were adjusted for each age group using pro-rata correction techniques for age at deaths. The sample size of deaths becomes smaller as the number of splits increases; therefore, modelling the age pattern of mortality is necessary to correct erratic points. The Gompertz-Makeham model was used to simulate the age pattern of mortality for each population subgroup starting at age 35 years and older [47]. Based on it, the present study constructed abridged life tables for India and states using Chiang's (1972) method [48].

Chiang method is based on the derivation of relation for the total number of person-years lived between exact ages x and x + n (${{}_{\mathrm{n}}\mathrm{L}}_{\mathrm{x}})$ in terms of the average number of years lived by an individual of age x who dies in the interval (x, x + n) $({{}_{\mathrm{n}}\mathrm{a}}_{\mathrm{x}}$). The following formulae are used to generate the columns of the life table:

${{}_{\mathrm{n}}\mathrm{q}}_{\mathrm{x}}:\mathrm{ the}$ probability of dying between age x and x + n

$${{}_{\mathrm{n}}\mathrm{q}}_{\mathrm{x}}=\frac{\mathrm{n}*({{}_{\mathrm{n}}\mathrm{M}}_{\mathrm{x}})}{1+(\mathrm{n}- {{}_{\mathrm{n}}\mathrm{a}}_{\mathrm{x}}) * {{}_{\mathrm{n}}\mathrm{M}}_{\mathrm{x}}}$$

${\mathrm{l}}_{\mathrm{x}}:$ number of people alive at the exact age x among a hypothetical birth cohort of 100,000, usually called the radix of the life table.

$${\mathrm{l}}_{\mathrm{x}+\mathrm{n}}={\mathrm{l}}_{\mathrm{x}}*\left(1-{{}_{\mathrm{n}}\mathrm{q}}_{\mathrm{x}}\right)$$

${{}_{\mathrm{n}}\mathrm{L}}_{\mathrm{x}}:$ total number of person-years lived between exact ages x and x + n

$${{}_{\mathrm{n}}\mathrm{L}}_{\mathrm{x}}=\mathrm{n}*({\mathrm{l}}_{\mathrm{x}}- {{}_{\mathrm{n}}\mathrm{d}}_{\mathrm{x}}+{{}_{\mathrm{n}}\mathrm{a}}_{\mathrm{x}}*{{}_{\mathrm{n}}\mathrm{d}}_{\mathrm{x}} )$$

${{}_{\mathrm{n}}\mathrm{d}}_{\mathrm{x}}:$ number of deaths in the age interval x to x + n

$${{}_{\mathrm{n}}\mathrm{d}}_{\mathrm{x}}={\mathrm{l}}_{\mathrm{x}}*{{}_{\mathrm{n}}\mathrm{q}}_{\mathrm{x}}$$

${\mathrm{T}}_{\mathrm{x}}:$ total number of person-years lived beyond Age x

$${\mathrm{T}}_{\mathrm{x}}={\mathrm{T}}_{\mathrm{x}+\mathrm{n}}+{{}_{\mathrm{n}}\mathrm{L}}_{\mathrm{x}}$$

${\mathrm{e}}_{\mathrm{x}}$: average number of years of life remaining for a person alive at the beginning of age interval x.

$${\mathrm{e}}_{\mathrm{x}}=\frac{{\mathrm{T}}_{\mathrm{x}}}{{\mathrm{l}}_{\mathrm{x}}}$$

#### Calculation of disparity in lifespan or inequality in age at death

For an abridged life table, The life disparity (${e}_{0}^{\dagger}$), a measure of the average number of life-years lost at birth, is estimated by

$${e}_{0}^{\dagger}=\frac{1}{{\mathrm{l}}_{\mathrm{x}}}{\sum }_{\mathrm{x}}^{\upomega }[{\mathrm{d}}_{\mathrm{x}}({\mathrm{e}}_{\mathrm{x}+1}+1-{\mathrm{a}}_{\mathrm{x}})]+\frac{{\mathrm{d}}_{\upomega }}{{\mathrm{l}}_{\upomega }}\left(\frac{{\mathrm{e}}_{\upomega }}{2}\right),$$

Where, ${\mathrm{e}}_{\mathrm{x}+1}$ is the remaining life expectancy at age x + 1, ${\mathrm{a}}_{\mathrm{x}}$ is the average person-years lived in an age interval by those who died in that age interval, and ${\mathrm{d}}_{\mathrm{x}}$ is life table deaths at age x.

## Results

### Variability in age pattern of mortality in India NFHS (2015–16, 2019–21) and SRS (2015, 2020)

**Fig. 1:**
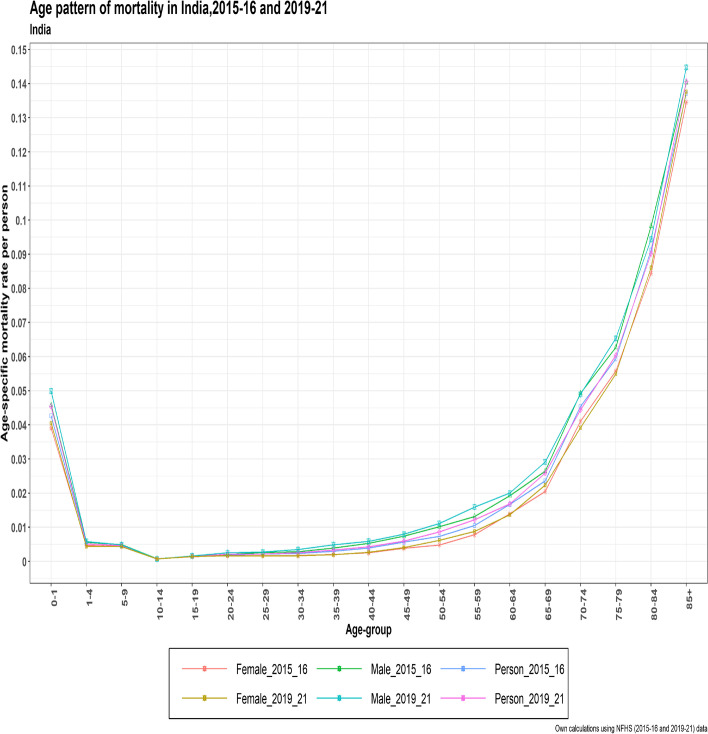
The age pattern of mortality in India for Person, male, and female from NFHS (2015–16) and NFHS (2019–21)

A higher age pattern of mortality was observed in the pandemic year 2019–21 as compared to the non-pandemic years 2015–16. In the adult and older age groups, a higher age pattern of mortality was observed in the pandemic years versus the non-pandemic years. Both datasets reflected a higher mortality for men than women (Fig. 1).

**Fig. 2:**
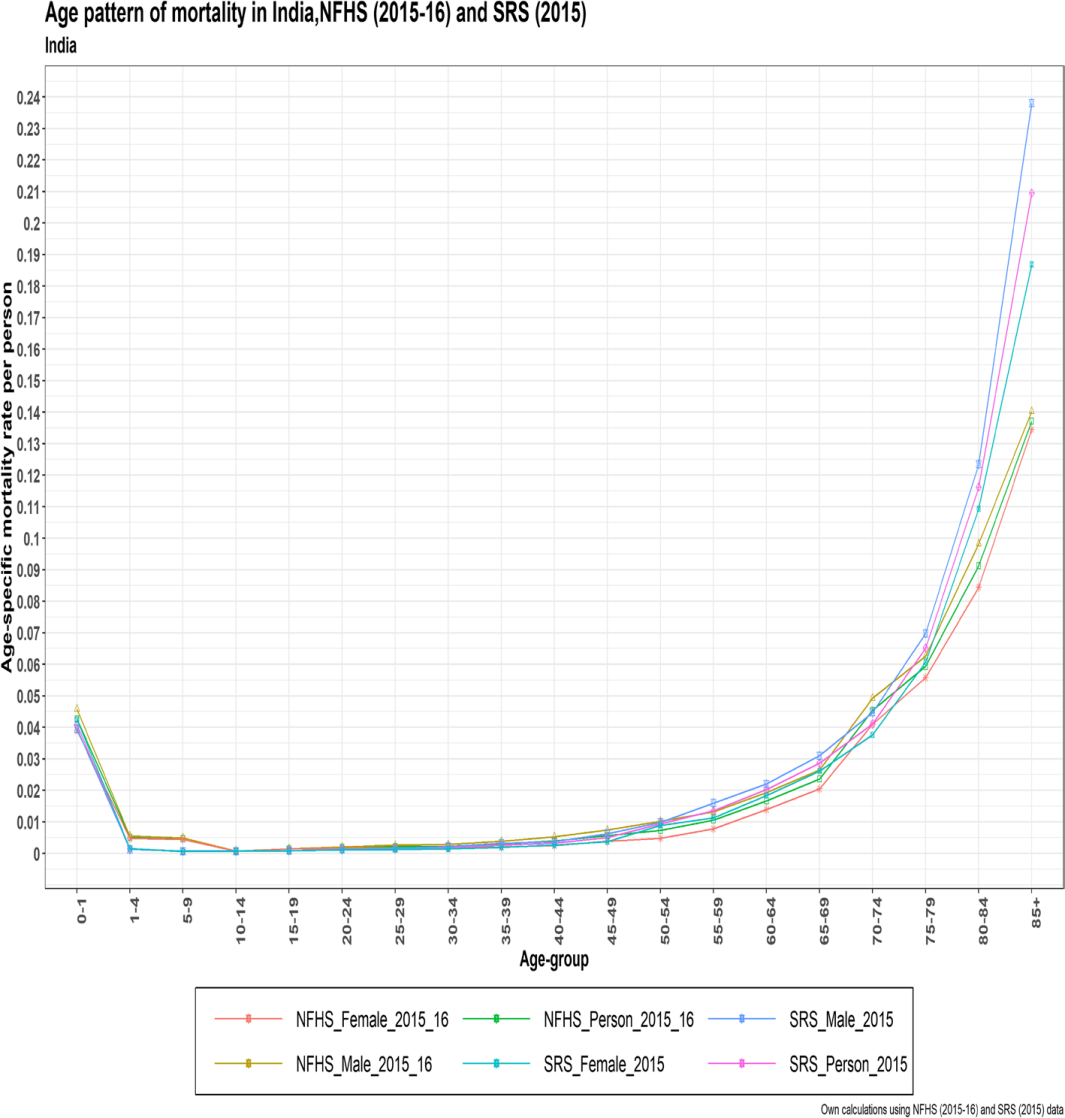
The age pattern of mortality in India for Person, male, and female from NFHS (2015–16) and SRS (2015)

Between the ages of 0 and 14 years, the mortality pattern in NFHS4 was higher than that in SRS 2015, however the patterns overlapped in both datasets for the 15–44 years age range. The 45–79 age groups exhibited a crossover pattern, while for the 80 + years age group, SRS 2015 demonstrated a higher age pattern of death than NFHS4. Male mortality was higher than female mortality in both datasets (Fig. 2).

**Fig. 3:**
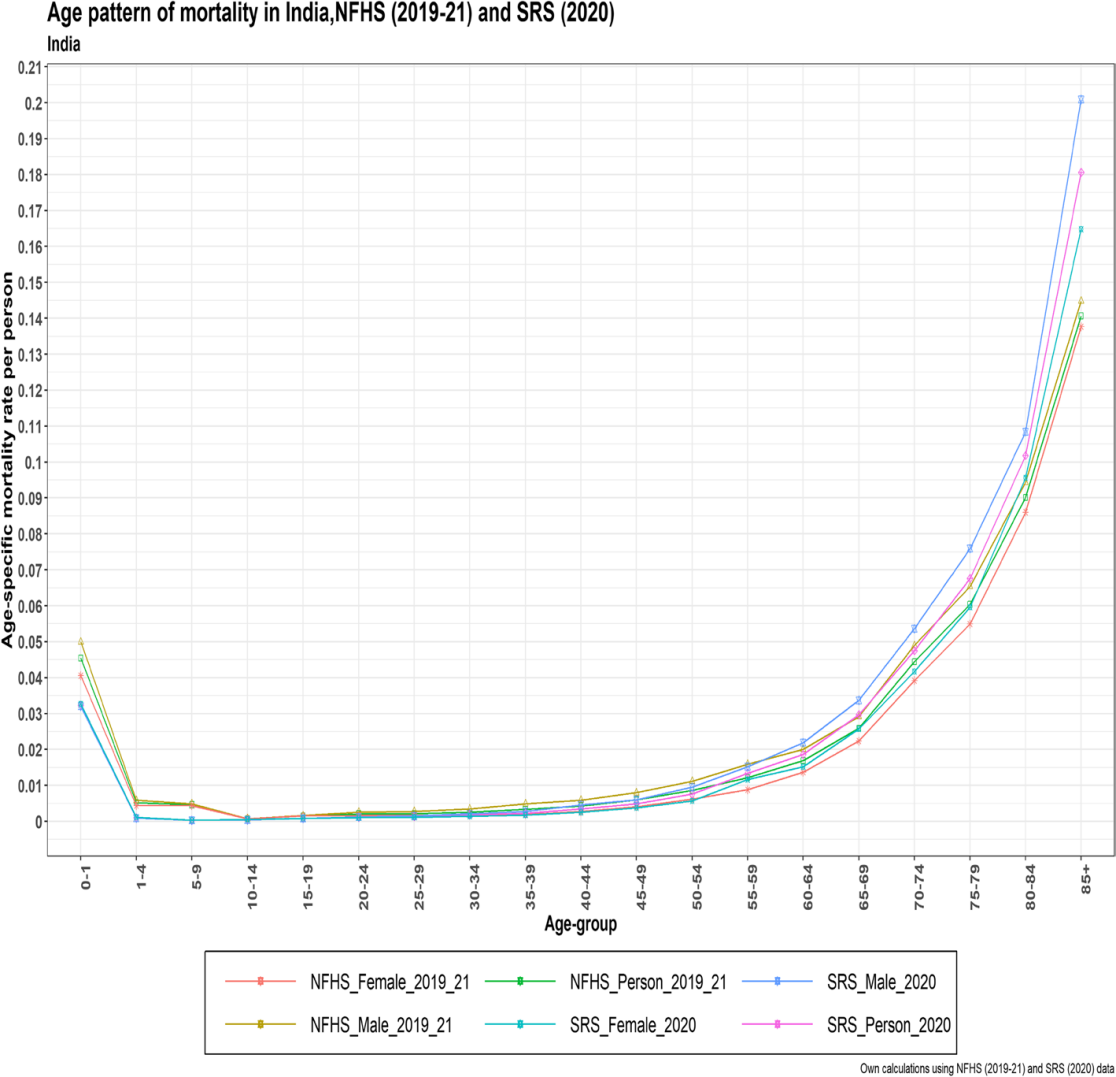
The age pattern of mortality in India for Person, male, and female from NFHS (2019–21) and SRS (2020)

While the mortality patterns at ages 10 to 29 years coincided in both datasets, the mortality patterns at ages zero to 14 were higher in NFHS5 than in SRS 2020. Crossover patterns predominated in the age ranges of 25 to 79 years, and for the 80–84 and 85 + years, SRS 2020 revealed a larger mortality pattern by age than NFHS5. Male mortality is higher than female mortality in both datasets (Fig. 3).

### Estimates of life expectancy and life disparity at birth in India, NFHS (2015–16, 2019–21) and SRS (2015, 2020)

In comparison to NFHS4, SRS 2015 showed higher life expectancy estimates among the ages 0–1, 1–4, and 5–9 years. Between the ages of 10 and 85 + , NFHS 4 demonstrated a longer life expectancy than SRS 2015. In each sets of data, women had longer life expectancies than males for each age group (Table 1).

**Table 1:** Life expectancy $({e}_{x})$ estimates for the person, male and Female in India NFHS (2015–16), SRS (2015)

Age-group	NFHS (2015–16)			SRS (2015)		
	**Person**	**Male**	**Female**	**Person**	**Male**	**Female**
**Below 1**	66.6	64.3	69.2	68.0	66.8	69.3
**1–4**	68.4	66.2	70.9	69.8	68.5	71.2
**5–9**	65.9	63.7	68.2	66.2	64.8	67.6
**10–14**	62.4	60.3	64.7	61.4	60.0	62.8
**15–19**	57.6	55.5	60.0	56.6	55.2	58.1
**20–24**	53.0	50.9	55.4	51.8	50.5	53.3
**25–29**	48.5	46.4	50.9	47.2	45.8	48.6
**30–34**	44.0	42.0	46.3	42.5	41.2	43.9
**35–39**	39.5	37.6	41.7	37.9	36.6	39.2
**40–44**	35.1	33.3	37.1	33.3	32.2	34.6
**45–49**	30.8	29.2	32.5	28.9	27.8	30.0
**50–54**	26.6	25.3	28.2	24.6	23.7	25.6
**55–59**	22.6	21.5	23.8	20.7	19.8	21.7
**60–64**	18.8	17.9	19.7	17.1	16.4	17.9
**65–69**	15.3	14.7	16.1	13.8	13.2	14.5
**70–74**	12.1	11.6	12.7	10.8	10.2	11.4
**75–79**	10.0	9.6	10.4	8.1	7.6	8.6
**80–84**	8.1	7.7	8.4	5.8	5.3	6.3
**85 + **	7.3	7.1	7.4	4.8	4.2	5.4

The SRS 2020 observed a longer life expectancy than the NFHS5 in the age ranges of 0 to 1 year, 1 to 4 years, and 5 to 9 years. Both datasets showed an overlapping pattern of life expectancy in the age range of 10 to 29 years. In the age range of 35 to 85 + , NFHS 5 showed a longer life expectancy than SRS 2020. In all age groups, both datasets demonstrate that women have a longer life expectancy than men (Table 2).

**Table 2:** Life expectancy $({e}_{x})$ estimates for the person, male and Female in India NFHS (2019–21), SRS (2020)

Age-group	NFHS (2019–21)			SRS (2020)		
	**Person**	**Male**	**Female**	**Person**	**Male**	**Female**
**Below 1**	65.8	62.9	68.9	69.3	67.6	71.2
**1–4**	67.8	65.1	70.7	70.5	68.8	72.5
**5–9**	65.1	62.6	67.9	66.8	65.0	68.8
**10–14**	61.6	59.1	64.4	61.9	60.1	63.9
**15–19**	56.9	54.3	59.6	57.0	55.2	59.0
**20–24**	52.3	49.7	55.1	52.3	50.5	54.3
**25–29**	47.8	45.4	50.5	47.6	45.8	49.5
**30–34**	43.3	41.0	45.9	42.9	41.1	44.8
**35–39**	38.9	36.7	41.3	38.2	36.5	40.1
**40–44**	34.5	32.6	36.7	33.7	32.0	35.5
**45–49**	30.3	28.5	32.2	29.2	27.7	30.9
**50–54**	26.1	24.6	27.8	24.9	23.6	26.5
**55–59**	22.3	21.0	23.6	20.9	19.7	22.2
**60–64**	18.6	17.6	19.7	17.3	16.2	18.5
**65–69**	15.1	14.4	16.0	13.9	12.9	14.9
**70–74**	12.1	11.5	12.8	10.9	10.1	11.8
**75–79**	9.9	9.4	10.3	8.6	7.9	9.3
**80–84**	8.0	7.7	8.3	6.7	6.1	7.2
**85 + **	7.1	6.9	7.3	5.5	5.0	6.1

NFHS4 estimates of life disparity were higher than SRS 2015 across all age groups. In comparison to SRS 2015, life disparity at birth ${e}_{0}^{\dagger}$ is 4.9 years longer for persons, 5.1 years longer for males, and 4.5 years longer for women in NFHS4. For all age categories, both datasets demonstrate that men have a higher life disparity than women (Table 3).

**Table 3:** Life disparity $({e}_{x}^{\dagger})$ Estimates for the person, male and Female in India NFHS (2015–16), SRS (2015)

Age-group	NFHS (2015–16)			SRS (2015)		
	**Person**	**Male**	**Female**	**Person**	**Male**	**Female**
**Below 1**	20.69	21.07	20.13	15.75	15.93	15.55
**1–4**	18.44	18.78	17.95	13.40	13.44	13.32
**5–9**	17.37	17.68	16.91	13.08	13.07	13.04
**10–14**	16.22	16.53	15.76	12.91	12.91	12.86
**15–19**	16.04	16.37	15.57	12.74	12.75	12.71
**20–24**	15.75	16.08	15.26	12.56	12.55	12.52
**25–29**	15.40	15.74	14.91	12.33	12.34	12.29
**30–34**	15.05	15.34	14.61	12.10	12.13	12.04
**35–39**	14.73	14.97	14.34	11.85	11.93	11.74
**40–44**	14.37	14.54	14.07	11.53	11.68	11.36
**45–49**	13.97	14.05	13.79	11.21	11.40	11.01
**50–54**	13.50	13.49	13.44	10.81	11.09	10.52
**55–59**	13.03	12.88	13.10	10.20	10.48	9.92
**60–64**	12.52	12.30	12.68	9.53	9.90	9.16
**65–69**	11.96	11.70	12.17	8.79	9.20	8.39
**70–74**	11.48	11.21	11.71	8.08	8.53	7.64
**75–79**	10.93	10.67	11.16	7.49	7.97	7.01
**80–84**	10.65	10.44	10.83	7.12	7.63	6.62
**85 + **	10.48	10.27	10.66	6.92	7.38	6.46

At all ages, NFHS5 estimates of life disparity were higher than those in SRS 2020. In NFHS5 compared to SRS 2020, life disparity at birth (${e}_{0}^{\dagger}$ ) is 5 years higher for persons, 5.5 years higher for men, and 4.6 years higher for women. At all ages, both datasets demonstrate greater life disparity for males than for women (Supplementary Table S1).

### Comparison of ${e}_{0}$ and ${e}_{0}^{\dagger}$ estimates for the person, male and female in India and the states from NFHS (2015–16, 2019–21) and SRS (2015, 2020)

#### Subnational variation in life expectancy at birth $({e}_{0})$ from NFHS (2015–16) and SRS (2015)

The highest and lowest ${e}_{0}$ estimates were observed for women in Mizoram 81 years and men in Telangana 58.4 years respectively in NFHS 4. SRS 2015 estimates found the highest and lowest ${e}_{0}$ of 77.6 years and 62.8 years for Jammu Kashmir women and Chhattisgarh men respectively. North region estimates observed the highest and lowest ${e}_{0}$ of 79.5 years and 64.1 years among Chandigarh women and Uttarakhand men in NFHS 4 and 77.6 years and 64.9 years in Jammu Kashmir women and Rajasthan men in SRS 2015 respectively. The ${e}_{0}$ estimates for the central region are highest for women from Madhya Pradesh at 68.8 years in NFHS 4 and 66.3 years in SRS 2015, while the lowest ${e}_{0}$ was for men from Uttar Pradesh at 63.3 years in NFHS 4 and for men from Chattisgarh at 62.8 years in SRS 2015. In the estimates for the eastern region, the highest ${e}_{0}$ was observed among women from West Bengal at 69.2 years in NFHS4 and 71.5 years in SRS 2015, respectively, and the lowest ${e}_{0}$ was observed among men from Bihar at 62 years in NFHS4 and men from Odisha at 65.8 years in SRS 2015. The estimates for the North Eastern region showed the highest and lowest ${e}_{0}$ for women in Mizoram (81 years) and men in Assam (61 years) in NFHS 4, and 66.4 years for women in Assam and 64.5 years for men in Assam in SRS 2015. The ${e}_{0}$ estimates for the western region were highest and lowest for women in Goa (74 years) and for men in Daman Diu (61.7 years) in NFHS 4, and women in Maharashtra (72.7 years) and men in Gujarat (66.6 years) in SRS 2015. In the southern region estimates, the highest ${e}_{0}$ was observed among women in Kerala at 77.8 years in NFHS4 and 77.2 years in SRS 2015, while the lowest ${e}_{0}$ was observed among men in Telangana (58.4 years) in NFHS4 and men in Karnataka (66.8 years) in SRS 2015 (Supplementary Table S2).

#### Subnational variation in life expectancy at birth $({e}_{0})$ from NFHS (2019–21) and SRS (2020)

Supplementary Table S3 shows the estimates of ${e}_{0}$ from NFHS (2019–21) and SRS (2020) at national and subnational level in India. The highest and lowest estimates of ${e}_{0}$ across 36 states were observed for Andaman and Nicobar Islands women (84.3 years) and Puducherry men (59 years) in NFHS 5 respectively, while SRS 2020 estimations revealed the highest ${e}_{0}$ in Jammu Kashmir women (80.3 years) and the lowest ${e}_{0}$ in Chhattisgarh men (62 years) across the 22 states. ${e}_{0}$ estimates in the north region observed the highest for Rajasthan women 75.4 years in NFHS 5 and Jammu and Kashmir women 80.3 years in SRS 2020, however the lowest ${e}_{0}$ was observed for Haryana men of 61.9 years in NFHS 5 and 65.3 years in SRS 2020 respectively. Central region ${e}_{0}$ estimates were observed highest 69.5 years in NFHS 5 and 69.1 years in SRS 2020 for Madhya Pradesh women and the lowest ${e}_{0}$ was observed for Uttar Pradesh men (60.6 years) in NFHS 5 and Chhattisgarh women (62 years) in SRS 2020. From NFHS5 the eastern region ${e}_{0}$ estimates were maximum for women (67.1 years) and lowest for males (61.4 years) in Odisha. However, from SRS 2020 the eastern region ${e}_{0}$ estimates were highest and lowest for women from West Bengal (73.4 years) and Jharkhand (68.1 years) respectively. In NFHS 5 Nagaland women (79.9 years) and Sikkim men (62.7 years) had the highest and lowest values of the northeast region ${e}_{0}$ respectively, however SRS 2020 estimates reveal that Assam women (67.9 years) and men (66.6 years) have the highest and lowest ${e}_{0}$ respectively. Western region ${e}_{0}$ estimates observed the highest and lowest for Goa women (79.1 years) and Gujarat men (64.3 years) in NFHS 5 respectively, while SRS 2020 estimates show the highest ${e}_{0}$ in Maharashtra women (74.2 years) and the lowest ${e}_{0}$ in Gujarat men (67.7 years) respectively. In the southern region estimates highest ${e}_{0}$ was observed in Andaman and Nicobar Islands women 84.3 years and the lowest ${e}_{0}$ of 59 years in Puducherry men in NFHS5, however SRS 2020 estimates show the highest ${e}_{0}$ of 77.1 years in Kerala women and the lowest ${e}_{0}$ of Karnataka men of 66.6 years respectively.

#### Subnational variation in life disparity at birth $({e}_{0}^{\dagger})$ from NFHS (2015–16) and SRS (2015)

Supplementary Table S4 shows the life disparity at birth (${e}_{0}^{\dagger}$) at the subnational level in India across 36 states from NFHS (2015–16) and 22 states from SRS (2015). Women from Mizoram and Lakshadweep observed the highest and lowest ${e}_{0}^{\dagger}$ of 35.4 years and 14.5 years respectively in NFHS4, SRS 2015 estimates observed the highest and lowest ${e}_{0}^{\dagger}$ in Jammu Kashmir men (18.8 years) and Delhi women (12.1 years) respectively across states and in the north. North region estimates show the highest ${e}_{0}^{\dagger}$ in Himachal Pradesh men (23.4 years) and the lowest ${e}_{0}^{\dagger}$ in Delhi men (15.5 years) in NFHS4. Central region Estimates of ${e}_{0}^{\dagger}$ was observed highest and lowest for Uttar Pradesh men (21 years) and Chhattisgarh women (19.5 years) in NFHS 4, however SRS 2015 estimates observed the highest and lowest ${e}_{0}^{\dagger}$ in Uttar Pradesh men (17.5 years) and Chattisgarh women (14.2 years). Life disparity at birth ${e}_{0}^{\dagger}$ was highest among Odisha men (22.2 years) and lowest among West Bengal women (18 years) in NFHS4, however the SRS 2015 results found highest and lowest ${e}_{0}^{\dagger}$ in Odisha men (17 years) and Jharkhand men (13.9 years) in the eastern region. Northeast region ${e}_{0}^{\dagger}$ estimates observed the highest and lowest for women in Mizoram (35.4 years) and Tripura (18.8 years) respectively in NFHS 4 and SRS 2015 estimates shows the highest and lowest ${e}_{0}^{\dagger}$ for men (17.1 years) and women (16 years) in Assam respectively. ${e}_{0}^{\dagger}$ Estimates in the western region observed the highest and lowest for Goa men (21.4 years) and Dadra and Nagar haveli men (15.7 years) in NFHS 4, however SRS 2015 shows the highest and lowest ${e}_{0}^{\dagger}$ for Gujarat men (16.7 years) and Maharashtra men (14.5 years) respectively. South region estimates of ${e}_{0}^{\dagger}$ showed the highest and lowest in Tamil Nadu men (25.2 years) and Lakshadweep women (14.5 years) in NFHS4, however SRS 2015 estimates observed the highest and lowest ${e}_{0}^{\dagger}$ in women from Andhra Pradesh (16.5 years) and Kerala (12.8 years) respectively.

#### Subnational variation in life disparity at birth $({e}_{0}^{\dagger})$ from NFHS (2019–21) and SRS (2020)

Supplementary Table S5 shows the subnational level ${e}_{0}^{\dagger}$ estimates for the pandemic years from NFHS (2019–21) and SRS 2020. ${e}_{0}^{\dagger}$ Estimates have variability in both data sets, NFHS5 has a higher ${e}_{0}^{\dagger}$ than SRS 2020 at the national and sub-national level. The disparity differentials in both data sets were due to variations in age patterns of mortality. Men have higher disparity estimates than women in both datasets with some exceptions for few states. NFHS 5 estimates found the Andaman and Nicobar Island women have maximum ${e}_{0}^{\dagger}$ of 52.7 years and Goa women have minimum ${e}_{0}^{\dagger}$ of 12.8 years across 36 states and SRS 2020 shows the maximum and minimum ${e}_{0}^{\dagger}$ in Jammu Kashmir men were 23.3 years and Delhi women 12.3 years respectively in the north region and across 22 states. Northern states ${e}_{0}^{\dagger}$ observed the highest and lowest estimates in Himachal Pradesh men (23.1 years) and Chandigarh women (17.6 years) in NFHS 5. Central region ${e}_{0}^{\dagger}$ estimates observed the highest and lowest in Chhattisgarh men 22.6 years and Madhya Pradesh women 18.9 years however, SRS 2020 estimates observed the highest and lowest ${e}_{0}^{\dagger}$ in Uttar Pradesh men (17.5 years) and Chhattisgarh women (14.2 years) respectively. The eastern region showed the highest and lowest ${e}_{0}^{\dagger}$ of 21.3 years 18.1 years among Odisha men and Jharkhand women in NFHS5 whereas women from Odisha and Bihar shows the highest and lowest ${e}_{0}^{\dagger}$ of 17.3 years and 13.3 years respectively in SRS 2020. Northeast region estimates observed that Assam women show the lowest ${e}_{0}^{\dagger}$ of 18 years in NFHS5 and 16.8 years in SRS 2020 whereas Nagaland women have the highest ${e}_{0}^{\dagger}$ of 35.5 years in NFHS5 and Assam men have 18.2 years in SRS 2020 respectively. Western region ${e}_{0}^{\dagger}$ shows the highest and lowest estimates for dadra nagar haveli/Daman Diu men 39.5 years and Goa women 12.8 years in NFHS5, however ${e}_{0}^{\dagger}$ stimates from SRS 2020 show the highest and lowest ${e}_{0}^{\dagger}$ in Gujarat men (15.8 years) and Maharashtra men (14.5 years) respectively. The highest and lowest estimated ${e}_{0}^{\dagger}$ are 52.7 years for women in Andaman Nicobar Islands and 17.9 years in Andhra Pradesh in NFHS5, However SRS 2020 estimates showed 17.3 years in Andhra Pradesh women and 12.9 years in Kerala men respectively.

### Differentials in ${e}_{0}$ and ${e}_{0}^{\dagger}$ estimates in India and states between NFHS (2015–16 to 2019–21) and SRS (2015–2020)

#### Subnational differentials in life expectancy at birth $({e}_{0})$ from NFHS (2015–16, 2019–21) and SRS (2015, 2020)

Supplementary Table S6 shows the differentials in ${e}_{0}$ at national and subnational level for 36 states from NFHS and 22 states from SRS. The positive and negative differentials depicts the decrease and increase in ${e}_{0}$ for the respective states. The results depicts the positive change in ${e}_{0}$ across most of the states in the NFHS data and some of the states in the SRS data. NFHS observed a decline of 1.4 years among men and 0.3 years among women at the national level. North region estimates observed the maximum decline in ${e}_{0}$ for Chandigarh women (10 years) in NFHS and Punjab men (2.9 years) in SRS. Central region ${e}_{0}$ observed the maximum decline in Chhattisgarh men of 3.8 years in NFHS and 0.8 years in SRS and an increase in Madhya Pradesh women of 0.7 years in NFHS and 2.8 years in SRS. East region ${e}_{0}$ shows the maximum decline and increase in ${e}_{0}$ for person in West Bengal (4.4 years) and Bihar (1.4 years) in NFHS and SRS estimates observed the maximum decline and increase among men in ${e}_{0}$ for West Bengal (0.2 years) and Odisha (2.7 years). Men in Sikkim exhibit the greatest fall in ${e}_{0}$ of 6.7 years, and women in Arunachal Pradesh exhibit the greatest gain in ${e}_{0}$ of 3.6 years in the northeast region of NFHS. Men and women from the northeastern region of Assam have a rise and fall in ${e}_{0}$ of 2.1 years and 1.5 years, respectively, in SRS. Western region ${e}_{0}$ observed the most fall in ${e}_{0}$ of 5.7 years in Dadara and Nagar Haveli men and a maximum increase in Daman and Diu men of 6 years in NFHS, while SRS results observed the maximum decline in ${e}_{0}$ for Maharashtra men 0.4 years and maximum increase in ${e}_{0}$ of 2.2 years in Gujarat women. Southern region estimates of ${e}_{0}$ shows a maximum increase for Andaman and Nicobar Islands women (12.7 years) and a maximum decline for Puducherry women of 3.3 years in NFHS whereas SRS 2020 estimates found Kerala men (1.7 years) and Tamil Nadu women (3.5 years) have the maximum decrease and increase in ${e}_{0}$ respectively.

#### Subnational Differentials in life disparity at birth $({\mathrm{e}}_{0}^{\dagger})$ from NFHS (2015–16, 2019–21) and SRS (2015, 2020)

Supplementary Table S7 shows the national and subnational changes in ${\mathrm{e}}_{0}^{\dagger}$ from NFHS (2015–16, 2019–21) and SRS (2015, 2020) for 36 states and 22 states respectively. The positive and negative differentials show a decrease and increase in ${e}_{0}^{\dagger}$ respectively. National level results observed an increase in ${e}_{0}^{\dagger}$ for men by 0.3 years and a decrease in ${e}_{0}^{\dagger}$ for women by 0.1 years in NFHS, whereas SRS showed an increase in ${e}_{0}^{\dagger}$ of 0.05 years and a decrease in ${e}_{0}^{\dagger}$ of 0.10 years among men. North region estimates observed the highest increase and decrease in ${e}_{0}^{\dagger}$ for Punjab men (3.7 years) and Chandigarh women (4.5 years) in NFHS whereas the SRS observed for Delhi men (5.1 years) and Himachal Pradesh women (2.3 years) respectively. In the central region, ${e}_{0}^{\dagger}$ has increased by 2.8 years among Chhattisgarh men and declined by 1.8 years among Madhya Pradesh women in NFHS, however SRS shows ${e}_{0}^{\dagger}$ has declined maximum among Madhya Pradesh men (0.5 years) and Chhattisgarh men show the maximum increase in ${e}_{0}^{\dagger}$ of 0.6 years. Eastern region estimates observed the maximum decline and increase in ${e}_{0}^{\dagger}$ for West Bengal men (1 year) and Jharkhand women (1.6 years) in NFHS; however, SRS estimates observed the maximum decline and increase for Bihar women (1.9 years) and Jharkhand men (1.2 years). Northeast region ${e}_{0}^{\dagger}$ observed the maximum increase for Sikkim women (8.7 years) and maximum decrease for Mizoram women (7.8 years) in NFHS, while SRS showed the maximum decline and increase for women (0.4 years) and men (2.1 years) in Assam. SRS estimates observed the maximum decline in ${e}_{0}^{\dagger}$ of 0.9 years in Gujarat men in the western region and in the southern region, maximum decline and increase in ${e}_{0}^{\dagger}$ are observed in Telangana men (1.1 years) and Tamil Nadu women (2.9 years) respectively.

## Discussion

This study examines the subnational variation in ${e}_{0}$ and ${e}_{0}^{\dagger}$ in India and all 36 states for persons, males, and females for NFHS (2015–16, 2019–21) and SRS (2015, 2020). The findings clearly show that ${e}_{0}$ and ${e}_{0}^{\dagger}$ vary across the India and between states. Moreover, the findings also demonstrates that ${e}_{0}$ and ${e}_{0}^{\dagger}$  are very strongly correlated, and the phenomenon of high ${e}_{0}$ and low ${e}_{0}^{\dagger}$ holds true for India and its all 36 states. This study discovered in common with the previous findings that the Covid-19 disease has disproportionally affected ${e}_{x}$ among men and women at the national and subnational level with significant regional differences between states in India. This demonstrates that by looking at inequality in life expectancy, more can be learned than life expectancy about the mortality situations in the Indian population.${e}_{0}$ has declined among men by 1.4 years and 0.3 years among women; similarly, it has declined for the 22 states in total, 23 states in men, and 22 states in women between 2015–16 and 2019–21 in the Indian population as per the NFHS results. According to SRS estimates, ${e}_{0}$ has declined for 7 states in total, 9 states in men and 5 states in women in India. Our findings show from NFHS estimates that ${e}_{0}^{\dagger}$ has increased for 21 states in person, 24 states in men, and 17 states in women between 2015–16 and 2019–21 among the 36 Indian states. Similarly, SRS estimates show ${e}_{0}^{\dagger}$ has increased for 11 states in Total, 9 states in men, and 10 states in women among the 22 Indian states during 2015–2020. A similar research study found that ${e}_{0}$ halted in the US black population and life span disparity increased in six states between 1980 and 1990 due to increased mortality from HIV [31]. Our findings show that men are more affected by the recent Covid-19 pandemic and lost 1.4 years in ${e}_{0}$ between 2015–16 to 2019–21 at the national level and ${e}_{0}$ has decreased for 23 states and ${e}_{0}^{\dagger}$ has increased for 24 states. The study by Yadav et al. 2021 shows that the Covid-19 pandemic has larger dispersion in age at death and depicts higher heterogeneity in the mortality pattern of Covid-19 disease, more strongly among men than women [19, 49, 50]. We found that at the national level ${e}_{0}$ shows a drop of 0.8 years ≈ 1 year between 2015–16 and 2019–21. Similar research findings show that ${e}_{0}$ has dropped by 2 years in the pandemic year 2020 [19] and 2.6 years in the pandemic year 2021 due to increased mortality from Covid-19 disease [26]. Our study found that ${e}_{0}$ has declined for 22 states out of a total of 36 Indian states. Similar findings were obtained in a population-level study of 29 countries examining the impacts of the Covid-19 pandemic on ${e}_{0}$ shows ${e}_{0}$ has declined from 2019 to 2020 in 27 out of 29 countries. Reductions in ${e}_{0}$ were mostly attributable to increased mortality above age 60 years and to official COVID-19 deaths [25].

According to a study in Brazil, ${e}_{0}$ shows a decline of 1.3 years in 2020, reaching a level not seen since 2004. The ${e}_{0}$ among males fall was greater, extending the male–female gap in ${e}_{0}$ by 9.1% [23]. In 2020, 31 countries lost more than 28 million additional years of life, with men having a higher rate than women. Compared to the seasonal influenza pandemic in 2015, the excess years of life lost due to the COVID-19 pandemic in 2020 was more than five times greater [51]. In 2020, the Covid-19 pandemic reduced US ${e}_{0}$ by 1.31 years. These reductions in ${e}_{0}$ are 3.2 times higher for the Latino population (${e}_{0}=$ 3.03) and two times larger for the black population (${e}_{0}=$ 1.90) as compared to the white population (${e}_{0}=$ 0.94) [52]. Therefore, the recent Covid-19 pandemic caused significant mortality increases in the pandemic year 2019–21 compared to the base year 2015–16. Women from 22 states and men from 23 states had a lower ${e}_{0}$ in 2019–21 than in 2015–16. Results from NFHS data had observed the higher life disparity compared to SRS based results. NFHS has a higher mortality in the ages 0-14 years and lower mortality beyond the age-group 75-79 years compared to the SRS. At certain age groups NFHS has lower life expectancy compared to SRS and lower life expectancy results in higher life disparity as both the measures are negatively correlated [53]. This would be the possible reason for higher life disparity in NFHS compared to SRS data. This finding is similar to the earlier studies which have found that, even in societies with comparable levels of life expectancy, different levels of life disparity can be observed [39, 54, 55]. Based on life tables for 212 nations, a 2009 study by Smits & Monden found that countries that hit a certain level of life expectancy earlier in time were more likely to experience higher levels of inequality [39]. However, the findings of a study by Seaman et al. (2016) are contradictory: Scotland, which had caught up to England and Wales in terms of life expectancy, had bigger variations in lifespan due to its lower mortality among older adult populations but greater premature mortality among adults [54].

The First case of COVID-19 pandemic was reported on 30 January 2020 in India [56] and since then country has seen 3 waves of infection and 5.3 lakhs deaths across India [57]. However, in early April 2021, a second major wave of infection began with devastating consequences [58]; on 9 April, India passed the 1 million active cases mark, and on 12 April, India overtook Brazil as the country with the second most COVID -19 cases worldwide [59]. By the end of April, India passed the 2.5 million active cases mark, reporting an average of 300,000 new cases and 2,000 deaths per day. Some analysts feared that this was under-reporting. On 30 April, India reported over 400,000 new cases and over 3,500 deaths in one day [60]. The present study investigated the variation in life expectancy and life disparity at national and subnational level in India. This study calculated ${e}_{0}$ and ${e}_{0}^{\dagger}$ before the pandemic from NFHS (2015–16), SRS (2015) and during the COVID-19 pandemic from NFHS (2019–21), SRS (2020). The NFHS-5 (2019–21) fieldwork for India was conducted in two phases, phase one from 17 June 2019 to 30 January 2020 and phase two from 2 January 2020 to 30 April 2021 by field agencies and collected information on deaths of a common household member from January 2016 to April 2021 and collected the deaths occurred in the pandemic year 2020–21 for India and 36 states. Similarly, SRS (2020) provides the age specific death rates for India and 22 states in the pandemic year 2020. Therefore, two recent surveys NFHS (2019–21) and SRS (2020) have collected the information on deaths due to COVID-19 pandemic. Major strengths of the study is that it calculated ${e}_{0}$ and ${e}_{0}^{\dagger}$ for all the 36 Indian States in the pandemic year 2019–21 and non-pandemic year 2015–16. In general, the life expectancy at birth in India has increased over the time and a recent study by Yadav et al. (2021) has shown that India's ${e}_{0}$ in the pandemic year 2020 decreased by 2.0 years compared to the non-pandemic year 2019. Another study aimed at tracking ${e}_{0}$ losses globally found that Indians lost 2.6 years in their ${e}_{0}$ in 2021 due to the COVID-19 pandemic [10]. These studies strengthen the inference that losses in ${e}_{0}$ at national and subnational level in India in the pandemic year 2019–21 from both data sources NFHS (2019–21) and SRS (2020) may have occurred due to COVID-19 pandemic alone and not due to other causes of mortality.

### Limitations of the study

While this study is one of the early works of its kind to look into the subnational variations in life expectancy and life disparity at birth in the context of COVID-19 pandemic, it does have some limitations. Firstly, since this is an actual cross-sectional evidence-based study that can only infer about period life expectancy and life disparity estimates and not the cohort based life expectancy and life disparity figures. Second, there has been used two data sources NFHS and SRS, the information available on age pattern of mortality in both the sources are different. NFHS gives information on age specific mortality rates for all the 36 states, which comprises of 28 states and 8 union territories, whereas SRS gives information on age specific mortality rates for the 22 states. Therefore, this study can compare the estimates of life expectancy and life disparity for the 22 states from both the data sources and it could not perform the analysis for remaining 14 states from the SRS data because of unavailability of the data. Third, due to data limitations only age and sex specific information on age pattern of mortality were included in the analysis without looking into causes of deaths. Future health programmes may benefit more from the age specific mortality information separated into both age and cause. Fourth, the confirmed and deceased cases of COVID-19 pandemic is available at the subnational level but ASMR calculated at the state level are inaccurate due to large number of missing information on age-sex pattern of mortality about many cases.

## Conclusion

The estimates of ${e}_{0}$ and ${e}_{0}^{\dagger}$ show noticeable variations for the person, male and female populations across the states from NFHS (2015–16,2019–21) and SRS (2015,2020), respectively. The study found that ${e}_{0}$ has declined for most states while life disparity increased between 2015–16 and 2019–21. ${e}_{0}$ has declined more among men than among women. Over the whole period of 2015–21, in summary, on average, Covid-19 cost men 1.4 years in ${e}_{0}$, whereas the corresponding setback for women was only 0.3 years. Similarly, life disparity for men increased by 0.31 years, and for women decreased by 0.13 years from 2015–21. Covid-19 affected the average for 22 states for ${e}_{0}$ and 21 states for ${e}_{0}^{\dagger}$ for the whole period of 2015–21.

### Supplementary Information

**Additional file 1: Supplementary Table S1. **e_x^†  Estimates for Persons, male, and Female, in India NFHS (2019-21), SRS (2020). **Supplementary Table S2. **e_0   Estimates for Persons, male and Female, in India and states, NFHS (2015-16), SRS (2015). **Supplementary Table S3. e_0  **Estimates for Persons, male and Female, in India and states, NFHS (2019-21), SRS (2020). **Supplementary Table S4. **e_0^†  Estimates in India and states NFHS-4 (2015-16) and SRS (2015).** Supplementary Table S5. **e_0^†  Estimates in India and states NFHS-5(2019-21) and SRS (2020). **Supplementary Table S6. **Change in e_0 in India and states NFHS-4 (2015-16) to NFHS-5 (2019-21) and SRS (2015) to SRS (2020).** Supplementary Table S7. **Change in e_0^†  in India and states NFHS-4 (2015-16) to NFHS-5 (2019-21) and SRS (2015) to SRS (2020). 

## Data Availability

The datasets used in the current study are available on the DHS program portal at https://dhsprogram.com/data/available-datasets.cfm, and Office of the Registrar General & Census Commissioner, India (ORGI) at https://censusindia.gov.in/census.website/data/SRSSTAT.

## Abbreviations

NFHS: National Family Health Survey
COVID-19: Coronavirus disease of 2019
ORGI: Office of the Registrar General of India
ex: Life expectancy at age x
${e}_{x}^{\dagger}$: Life disparity at age x
${e}_{0}$: Life expectancy at birth
${e}_{0}^{\dagger}$: Life disparity at birth
NA: Not Applicable
