# Supplementary material for: Impact of COVID-19 on subnational variations in life expectancy and life disparity at birth in India: evidence from NFHS and SRS data
Source: Arch Public Health. 2023 Sep 4;81:165. doi: 10.1186/s13690-023-01170-8 (PMC10476359; doi:10.1186/s13690-023-01170-8)
Supplement: Supplementary file 1 — Additional file 1: Supplementary Table S1. e_x^† Estimates for Persons, male, and Female, in India NFHS (2019-21), SRS (2020). Supplementary Table S2. e_0 Estimates for Persons, male and Female, in India and states, NFHS (2015-16), SRS (2015). Supplementary Table S3. e_0 Estimates for Persons, male and Female, in India and states, NFHS (2019-21), SRS (2020). Supplementary Table S4. e_0^† Estimates in India and states NFHS-4 (2015-16) and SRS (2015). Supplementary Table S5. e_0^† Estimates in India and states NFHS-5(2019-21) and SRS (2020). Supplementary Table S6. Change in e_0 in India and states NFHS-4 (2015-16) to NFHS-5 (2019-21) and SRS (2015) to SRS (2020). Supplementary Table S7. Change in e_0^† in India and states NFHS-4 (2015-16) to NFHS-5 (2019-21) and SRS (2015) to SRS (2020). [file 13690_2023_1170_MOESM1_ESM.docx]

**Supplementary Tables**

| **Supplementary Table S1.** Life disparity$( e_{x}^{\dagger}$ ) estimates for Person, male and Female in India NFHS (2019-21), SRS (2020). | | | | | | |
| --- | --- | --- | --- | --- | --- | --- |
| **Age-group** | **NFHS (2019-21)** | | | **SRS (2020)** | | |
|  | **Person** | **Male** | **Female** | **Person** | **Male** | **Female** |
| **Below 1** | 20.82 | 21.38 | 20.00 | 15.76 | 15.83 | 15.60 |
| **1-4** | 18.46 | 18.95 | 17.74 | 13.87 | 13.86 | 13.78 |
| **5-9** | 17.41 | 17.84 | 16.79 | 13.65 | 13.60 | 13.58 |
| **10-14** | 16.30 | 16.74 | 15.66 | 13.57 | 13.52 | 13.51 |
| **15-19** | 16.14 | 16.59 | 15.49 | 13.46 | 13.40 | 13.42 |
| **20-24** | 15.83 | 16.30 | 15.16 | 13.29 | 13.22 | 13.26 |
| **25-29** | 15.45 | 15.88 | 14.83 | 13.08 | 13.03 | 13.04 |
| **30-34** | 15.11 | 15.48 | 14.55 | 12.87 | 12.84 | 12.81 |
| **35-39** | 14.76 | 15.05 | 14.30 | 12.61 | 12.62 | 12.53 |
| **40-44** | 14.37 | 14.53 | 14.05 | 12.35 | 12.40 | 12.22 |
| **45-49** | 13.95 | 14.01 | 13.75 | 12.00 | 12.13 | 11.79 |
| **50-54** | 13.47 | 13.43 | 13.39 | 11.60 | 11.80 | 11.34 |
| **55-59** | 12.92 | 12.80 | 12.95 | 11.13 | 11.41 | 10.79 |
| **60-64** | 12.34 | 12.12 | 12.49 | 10.49 | 10.81 | 10.13 |
| **65-69** | 11.78 | 11.51 | 11.98 | 9.87 | 10.25 | 9.46 |
| **70-74** | 11.25 | 10.97 | 11.46 | 9.23 | 9.64 | 8.80 |
| **75-79** | 10.71 | 10.43 | 10.92 | 8.60 | 9.02 | 8.15 |
| **80-84** | 10.40 | 10.13 | 10.60 | 8.16 | 8.62 | 7.68 |
| **85+** | 10.24 | 9.98 | 10.44 | 7.92 | 8.34 | 7.47 |

| **Supplementary Table S2.** Life expectancy at birth ($e_{0})$estimates for Person, male and Female in India and states, NFHS (2015-16), SRS (2015). | | | | | | |
| --- | --- | --- | --- | --- | --- | --- |
| **Region/State** | **NFHS (2015-16)** | | | **SRS (2015)** | | |
|  | **Person** | **Male** | **Female** | **Person** | **Male** | **Female** |
| **India** | **66.6** | **64.3** | **69.2** | **68.0** | **66.8** | **69.3** |
| **North** |  |  |  |  |  |  |
| Chandigarh | 73.3 | 69.1 | 79.5 | NA | NA | NA |
| Delhi | 69.2 | 66.8 | 73.1 | 74.6 | 72.9 | 76.5 |
| Haryana | 68.4 | 65.8 | 71.9 | 68.4 | 66.2 | 70.9 |
| Himachal Pradesh | 70.8 | 67.5 | 74.3 | 71.7 | 67.7 | 76.2 |
| Jammu and Kashmir | 69.2 | 67.6 | 70.8 | 74.4 | 72.0 | 77.6 |
| Punjab | 71.1 | 69.0 | 73.5 | 72.4 | 70.8 | 74.2 |
| Rajasthan | 70.2 | 67.3 | 73.4 | 67.0 | 64.9 | 69.4 |
| Uttarakhand | 67.6 | 64.1 | 71.6 | 69.2 | 66.4 | 72.2 |
| **Central** |  |  |  |  |  |  |
| Chhattisgarh | 66.4 | 64.6 | 68.3 | 64.3 | 62.8 | 65.9 |
| Madhya Pradesh | 66.0 | 63.6 | 68.8 | 64.5 | 62.9 | 66.3 |
| Uttar Pradesh | 64.9 | 63.3 | 66.6 | 63.9 | 63.4 | 64.3 |
| **East** |  |  |  |  |  |  |
| Bihar | 63.0 | 62.0 | 64.0 | 67.4 | 68.4 | 66.4 |
| Jharkhand | 66.7 | 64.9 | 68.6 | 67.4 | 67.4 | 67.4 |
| Odisha | 64.7 | 63.4 | 66.1 | 67.5 | 65.8 | 69.4 |
| West Bengal | 68.3 | 67.4 | 69.2 | 70.6 | 69.7 | 71.5 |
| **Northeast** |  |  |  |  |  |  |
| Arunachal Pradesh | 64.6 | 63.0 | 66.5 | NA | NA | NA |
| Assam | 63.5 | 61.0 | 66.4 | 65.4 | 64.5 | 66.4 |
| Manipur | 70.1 | 66.0 | 74.5 | NA | NA | NA |
| Meghalaya | 68.5 | 65.0 | 72.6 | NA | NA | NA |
| Mizoram | 72.1 | 66.1 | 81.0 | NA | NA | NA |
| Nagaland | 76.2 | 73.0 | 79.8 | NA | NA | NA |
| Sikkim | 69.8 | 69.4 | 70.7 | NA | NA | NA |
| Tripura | 67.8 | 64.4 | 71.8 | NA | NA | NA |
| **West** |  |  |  |  |  |  |
| Dadra and Nagar haveli | 73.0 | 73.4 | 72.7 | NA | NA | NA |
| Daman and diu | 66.2 | 61.7 | 72.5 | NA | NA | NA |
| Goa | 69.7 | 66.1 | 74.0 | NA | NA | NA |
| Gujarat | 68.7 | 65.9 | 72.0 | 68.9 | 66.6 | 71.4 |
| Maharashtra | 70.2 | 67.9 | 72.8 | 71.6 | 70.5 | 72.7 |
| **South** |  |  |  |  |  |  |
| Andaman and nicobar islands | 67.4 | 64.1 | 71.5 | NA | NA | NA |
| Andhra Pradesh | 64.2 | 59.9 | 69.2 | 69.0 | 67.8 | 70.3 |
| Karnataka | 68.2 | 64.6 | 72.3 | 68.2 | 66.8 | 69.4 |
| Kerala | 73.9 | 70.1 | 77.8 | 74.3 | 71.4 | 77.2 |
| Lakshadweep | 68.5 | 65.9 | 71.6 | NA | NA | NA |
| Puducherry | 66.3 | 60.3 | 73.9 | NA | NA | NA |
| Tamil Nadu | 63.6 | 60.2 | 67.4 | 70.4 | 68.5 | 72.4 |
| Telangana | 62.4 | 58.4 | 66.8 | 68.4 | 67.4 | 69.8 |

| **Supplementary Table S3.** Life expectancy at birth ($e_{0})$for Person, male and Female in India and states, NFHS (2019-21), SRS (2020). | | | | | | |
| --- | --- | --- | --- | --- | --- | --- |
| **Region/State** | **NFHS (2019-21)** | | | **SRS (2020)** | | |
|  | **Person** | **Male** | **Female** | **Person** | **Male** | **Female** |
| **India** | **65.8** | **62.9** | **68.9** | **69.3** | **67.6** | **71.2** |
| **North** |  |  |  |  |  |  |
| Chandigarh | 66.5 | 64.1 | 69.4 | NA | NA | NA |
| Delhi | 68.0 | 65.2 | 71.3 | 74.2 | 71.5 | 77.9 |
| Haryana | 66.0 | 61.9 | 71.0 | 68.2 | 65.3 | 71.7 |
| Himachal Pradesh | 70.6 | 67.9 | 73.2 | 71.7 | 68.5 | 75.7 |
| Jammu and Kashmir | 71.8 | 70.9 | 72.8 | 75.7 | 72.7 | 80.3 |
| Punjab | 66.2 | 63.3 | 69.4 | 69.9 | 67.8 | 72.2 |
| Rajasthan | 71.5 | 68.1 | 75.4 | 68.9 | 66.1 | 71.9 |
| Uttarakhand | 68.3 | 65.4 | 71.4 | 69.0 | 66.4 | 71.9 |
| **Central** |  |  |  |  |  |  |
| Chhattisgarh | 63.8 | 60.8 | 67.0 | 63.6 | 62.0 | 65.2 |
| Madhya Pradesh | 66.2 | 63.2 | 69.5 | 66.8 | 64.8 | 69.1 |
| Uttar Pradesh | 63.0 | 60.6 | 65.6 | 65.6 | 64.7 | 66.7 |
| **East** |  |  |  |  |  |  |
| Bihar | 64.4 | 63.3 | 65.4 | 68.6 | 68.7 | 68.5 |
| Jharkhand | 64.7 | 62.7 | 66.7 | 68.3 | 68.5 | 68.1 |
| Odisha | 64.2 | 61.4 | 67.1 | 69.6 | 68.5 | 71.0 |
| West Bengal | 64.7 | 63.0 | 66.6 | 71.3 | 69.5 | 73.4 |
| **Northeast** |  |  |  |  |  |  |
| Arunachal Pradesh | 67.5 | 65.0 | 70.2 | NA | NA | NA |
| Assam | 66.1 | 63.1 | 69.5 | 67.2 | 66.6 | 67.9 |
| Manipur | 67.6 | 63.7 | 71.7 | NA | NA | NA |
| Meghalaya | 68.8 | 66.5 | 71.4 | NA | NA | NA |
| Mizoram | 72.1 | 66.9 | 78.4 | NA | NA | NA |
| Nagaland | 75.4 | 71.5 | 79.9 | NA | NA | NA |
| Sikkim | 65.9 | 62.7 | 70.2 | NA | NA | NA |
| Tripura | 66.0 | 63.7 | 68.6 | NA | NA | NA |
| **West** |  |  |  |  |  |  |
| Dadra and Nagar haveli | 70.6 | 67.7 | 76.9 | NA | NA | NA |
| Daman and diu | 70.6 | 67.7 | 76.9 | NA | NA | NA |
| Goa | 73.3 | 68.7 | 79.1 | NA | NA | NA |
| Gujarat | 67.7 | 64.3 | 71.5 | 70.5 | 67.7 | 73.6 |
| Maharashtra | 69.3 | 66.4 | 72.4 | 72.0 | 70.1 | 74.2 |
| **South** |  |  |  |  |  |  |
| Andaman and nicobar islands | 67.1 | 62.1 | 84.3 | NA | NA | NA |
| Andhra Pradesh | 63.4 | 59.6 | 67.8 | 70.5 | 69.1 | 72.1 |
| Karnataka | 67.3 | 63.2 | 72.0 | 69.0 | 66.6 | 71.7 |
| Kerala | 71.2 | 67.1 | 75.5 | 73.3 | 69.7 | 77.1 |
| Lakshadweep | 71.2 | 66.1 | 76.3 | NA | NA | NA |
| Puducherry | 64.6 | 59.0 | 70.7 | NA | NA | NA |
| Tamil Nadu | 63.9 | 59.3 | 69.1 | 73.5 | 71.3 | 75.9 |
| Telangana | 63.8 | 60.4 | 67.3 | 69.3 | 67.8 | 70.9 |

| **Supplementary Table S4.** Life disparity at birth ($e_{0}^{\dagger})$ estimates for Person, male and Female in India and states NFHS-4 (2015-16) and SRS (2015). | | | | | | |
| --- | --- | --- | --- | --- | --- | --- |
| **State name** | **NFHS (2015-16)** | | | **SRS (2015)** | | |
|  | **Person** | **Male** | **Female** | **Person** | **Male** | **Female** |
| **India** | **20.69** | **21.07** | **20.13** | **15.75** | **15.93** | **15.55** |
| **North** |  |  |  |  |  |  |
| Chandigarh | 21.90 | 21.54 | 22.10 | NA | NA | NA |
| Delhi | 16.50 | 15.55 | 19.77 | 12.97 | 13.56 | 12.14 |
| Haryana | 19.71 | 19.74 | 19.43 | 16.83 | 16.90 | 16.83 |
| Himachal Pradesh | 22.50 | 23.41 | 21.29 | 16.87 | 15.65 | 17.52 |
| Jammu and Kashmir | 20.02 | 21.06 | 18.90 | 18.07 | 18.77 | 17.46 |
| Punjab | 18.80 | 19.22 | 18.20 | 17.31 | 17.25 | 17.34 |
| Rajasthan | 19.44 | 19.79 | 18.78 | 16.39 | 17.33 | 15.50 |
| Uttarakhand | 19.99 | 20.67 | 18.94 | 15.59 | 14.99 | 15.92 |
| **Central** |  |  |  |  |  |  |
| Chhattisgarh | 19.70 | 19.80 | 19.49 | 14.74 | 15.23 | 14.20 |
| Madhya Pradesh | 20.81 | 20.78 | 20.67 | 16.51 | 16.52 | 16.41 |
| Uttar Pradesh | 20.70 | 21.00 | 20.36 | 16.82 | 17.55 | 16.25 |
| **East** |  |  |  |  |  |  |
| Bihar | 20.70 | 20.96 | 20.47 | 14.89 | 14.63 | 15.21 |
| Jharkhand | 19.95 | 20.20 | 19.70 | 14.54 | 13.90 | 15.48 |
| Odisha | 21.16 | 22.23 | 20.08 | 16.86 | 16.99 | 16.73 |
| West Bengal | 18.40 | 18.79 | 17.97 | 15.00 | 15.09 | 14.88 |
| **North East** |  |  |  |  |  |  |
| Arunachal Pradesh | 27.16 | 26.86 | 27.85 | NA | NA | NA |
| Assam | 21.12 | 21.58 | 20.43 | 16.62 | 16.04 | 17.15 |
| Manipur | 23.79 | 25.06 | 22.10 | NA | NA | NA |
| Meghalaya | 23.04 | 26.41 | 20.36 | NA | NA | NA |
| Mizoram | 31.41 | 28.87 | 35.37 | NA | NA | NA |
| Nagaland | 32.35 | 33.39 | 31.12 | NA | NA | NA |
| Sikkim | 23.75 | 29.40 | 18.86 | NA | NA | NA |
| Tripura | 18.90 | 18.82 | 18.82 | NA | NA | NA |
| **West** |  |  |  |  |  |  |
| Dadra and Nagar haveli | 15.80 | 15.65 | NA | NA | NA | NA |
| Daman and diu | 20.24 | 20.18 | 19.35 | NA | NA | NA |
| Goa | 19.04 | 21.36 | 16.85 | NA | NA | NA |
| Gujarat | 21.21 | 21.16 | 20.91 | 16.40 | 16.68 | 15.90 |
| Maharashtra | 18.84 | 19.25 | 18.21 | 15.13 | 14.55 | 15.66 |
| **South** |  |  |  |  |  |  |
| Andaman and nicobar islands | 17.29 | 18.59 | 15.91 | NA | NA | NA |
| Andhra Pradesh | 22.42 | 23.90 | 20.39 | 16.46 | 16.41 | 16.47 |
| Karnataka | 23.17 | 23.17 | 22.54 | 13.57 | 13.30 | 13.84 |
| Kerala | 20.66 | 21.42 | 19.56 | 13.39 | 13.80 | 12.77 |
| Lakshadweep | 17.65 | 20.35 | 14.53 | NA | NA | NA |
| Puducherry | 22.04 | 21.41 | 24.36 | NA | NA | NA |
| Tamil Nadu | 23.76 | 25.19 | 21.91 | 13.72 | 13.98 | 13.64 |
| Telangana | 22.96 | 24.09 | 21.37 | 14.48 | 15.76 | 13.78 |

| **Supplementary Table S5.** Life disparity at birth ($e_{0}^{\dagger})$ for Person, male and Female in India and states NFHS-5(2019-21) and SRS (2020). | | | | | | |
| --- | --- | --- | --- | --- | --- | --- |
| **State name** | **NFHS (2019-21)** | | | **SRS (2020)** | | |
|  | **Person** | **Male** | **Female** | **Person** | **Male** | **Female** |
| **India** | **20.82** | **21.38** | **20.00** | **15.76** | **15.83** | **15.60** |
| **North** |  |  |  |  |  |  |
| Chandigarh | 19.85 | 21.53 | 17.60 | NA | NA | NA |
| Delhi | 19.00 | 19.19 | 18.58 | 15.08 | 18.65 | 12.32 |
| Haryana | 21.36 | 21.75 | 20.42 | 15.85 | 15.13 | 16.13 |
| Himachal Pradesh | 22.35 | 23.09 | 21.52 | 15.62 | 16.01 | 15.22 |
| Jammu and Kashmir | 20.65 | 19.99 | 22.23 | 20.15 | 23.27 | 18.57 |
| Punjab | 21.88 | 22.91 | 20.59 | 16.08 | 15.92 | 16.18 |
| Rajasthan | 19.77 | 19.66 | 19.55 | 15.76 | 16.63 | 14.84 |
| Uttarakhand | 20.26 | 22.91 | 18.00 | 15.48 | 15.05 | 15.65 |
| **Central** |  |  |  |  |  |  |
| Chhattisgarh | 22.25 | 22.56 | 21.79 | 14.89 | 15.83 | 14.19 |
| Madhya Pradesh | 20.46 | 21.39 | 18.90 | 16.19 | 16.03 | 16.23 |
| Uttar Pradesh | 20.99 | 21.14 | 20.79 | 16.73 | 17.49 | 16.09 |
| **East** |  |  |  |  |  |  |
| Bihar | 20.39 | 21.14 | 19.71 | 13.56 | 13.85 | 13.34 |
| Jharkhand | 19.59 | 21.09 | 18.06 | 15.44 | 15.10 | 15.87 |
| Odisha | 20.25 | 21.34 | 18.98 | 17.25 | 17.18 | 17.30 |
| West Bengal | 19.23 | 19.79 | 18.51 | 14.42 | 14.16 | 14.50 |
| **North East** |  |  |  |  |  |  |
| Arunachal Pradesh | 25.36 | 26.05 | 24.61 | NA | NA | NA |
| Assam | 20.06 | 21.95 | 17.97 | 17.38 | 18.16 | 16.77 |
| Manipur | 23.15 | 24.60 | 21.40 | NA | NA | NA |
| Meghalaya | 28.09 | 30.85 | 26.20 | NA | NA | NA |
| Mizoram | 29.70 | 30.77 | 27.60 | NA | NA | NA |
| Nagaland | 33.81 | 33.71 | 35.47 | NA | NA | NA |
| Sikkim | 26.43 | 26.09 | 27.55 | NA | NA | NA |
| Tripura | 23.59 | 23.73 | 23.33 | NA | NA | NA |
| **West** |  |  |  |  |  |  |
| Dadra and Nagar haveli | 25.95 | 39.47 | 22.58 | NA | NA | NA |
| Daman and diu | 25.95 | 39.47 | 22.58 | NA | NA | NA |
| Goa | 17.47 | 21.68 | 12.77 | NA | NA | NA |
| Gujarat | 21.70 | 22.03 | 20.90 | 15.84 | 15.81 | 15.46 |
| Maharashtra | 20.53 | 21.42 | 19.31 | 14.70 | 14.50 | 14.80 |
| **South** |  |  |  |  |  |  |
| Andaman and nicobar islands | 26.02 | 21.51 | 52.68 | NA | NA | NA |
| Andhra Pradesh | 19.56 | 20.64 | 17.95 | 16.58 | 15.85 | 17.33 |
| Karnataka | 21.51 | 22.11 | 20.39 | 14.21 | 13.51 | 14.54 |
| Kerala | 23.32 | 22.62 | 23.27 | 13.58 | 12.88 | 13.80 |
| Lakshadweep | 20.82 | 22.19 | 18.72 | NA | NA | NA |
| Puducherry | 25.42 | 29.04 | 21.26 | NA | NA | NA |
| Tamil Nadu | 21.92 | 22.94 | 20.38 | 15.78 | 14.87 | 16.53 |
| Telangana | 20.85 | 21.71 | 19.58 | 15.38 | 14.70 | 15.97 |

| **Supplementary Table S6.** Change in life expectancy at birth ($e_{0})$ estimates for Person, male and Female in India and states, NFHS-4 (2015-16) to NFHS-5 (2019-21) and SRS (2015) to SRS (2020). | | | | | | |
| --- | --- | --- | --- | --- | --- | --- |
| **Region/State** | **changes in** $\boldsymbol{e}_{\boldsymbol{0}}$**, NFHS, 2015-16 to 2019-21** | | | **changes in** $\boldsymbol{e}_{\boldsymbol{0}}$**, SRS 2015 to 2020** | | |
|  | **Person** | **Male** | **Female** | **Person** | **Male** | **Female** |
| **India** | 0.8 | 1.4 | 0.3 | -1.3 | -0.8 | -1.9 |
| **North** |  |  |  |  |  |  |
| Chandigarh | 6.9 | 5.0 | 10.1 | NA | NA | NA |
| Delhi | 1.2 | 1.7 | 1.8 | 0.4 | 1.5 | -1.4 |
| Haryana | 2.4 | 3.9 | 0.8 | 0.2 | 1.0 | -0.8 |
| Himachal Pradesh | 0.2 | -0.4 | 1.1 | 0.0 | -0.8 | 0.5 |
| Jammu Kashmir | -2.6 | -3.4 | -2.1 | -1.3 | -0.6 | -2.7 |
| Punjab | 4.9 | 5.6 | 4.1 | 2.5 | 2.9 | 2.0 |
| Rajasthan | -1.3 | -0.8 | -2.0 | -1.9 | -1.3 | -2.5 |
| Uttarakhand | -0.7 | -1.4 | 0.2 | 0.1 | 0.0 | 0.2 |
| **Central** |  |  |  |  |  |  |
| Chhattisgarh | 2.6 | 3.8 | 1.3 | 0.7 | 0.8 | 0.6 |
| Madhya Pradesh | -0.2 | 0.4 | -0.7 | -2.3 | -1.9 | -2.8 |
| Uttar Pradesh | 1.9 | 2.7 | 1.0 | -1.8 | -1.3 | -2.4 |
| **East** |  |  |  |  |  |  |
| Bihar | -1.4 | -1.3 | -1.3 | -1.2 | -0.2 | -2.1 |
| Jharkhand | 2.0 | 2.2 | 1.9 | -0.9 | -1.1 | -0.8 |
| Odisha | 0.6 | 2.1 | -1.1 | -2.2 | -2.7 | -1.5 |
| West Bengal | 3.5 | 4.4 | 2.6 | -0.7 | 0.2 | -1.9 |
| **Northeast** |  |  |  |  |  |  |
| Arunachal Pradesh | -2.9 | -2.0 | -3.6 | NA | NA | NA |
| Assam | -2.6 | -2.0 | -3.2 | -1.8 | -2.1 | -1.5 |
| Manipur | 2.5 | 2.2 | 2.8 | NA | NA | NA |
| Meghalaya | -0.3 | -1.5 | 1.2 | NA | NA | NA |
| Mizoram | 0.0 | -0.8 | 2.6 | NA | NA | NA |
| Nagaland | 0.8 | 1.5 | 0.0 | NA | NA | NA |
| Sikkim | 3.9 | 6.7 | 0.5 | NA | NA | NA |
| Tripura | 1.7 | 0.7 | 3.3 | NA | NA | NA |
| **West** |  |  |  |  |  |  |
| Dadara and Nagar Haveli | 2.4 | 5.7 | -4.2 | NA | NA | NA |
| Daman and Diu | -4.4 | -6.0 | -4.4 | NA | NA | NA |
| Goa | -3.6 | -2.6 | -5.1 | NA | NA | NA |
| Gujarat | 1.1 | 1.7 | 0.4 | -1.6 | -1.1 | -2.2 |
| Maharashtra | 1.0 | 1.5 | 0.4 | -0.5 | 0.4 | -1.5 |
| **South** |  |  |  |  |  |  |
| Andaman and nicobar islands | 0.3 | 2.0 | -12.7 | NA | NA | NA |
| Andhra Pradesh | 0.8 | 0.3 | 1.4 | -1.6 | -1.3 | -1.8 |
| Karnataka | 0.9 | 1.4 | 0.3 | -0.8 | 0.3 | -2.2 |
| Kerala | 2.7 | 3.0 | 2.4 | 1.0 | 1.7 | 0.2 |
| Lakshadweep | -2.7 | -0.2 | -4.7 | NA | NA | NA |
| Puducherry | 1.7 | 1.3 | 3.3 | NA | NA | NA |
| Tamil Nadu | -0.3 | 1.0 | -1.7 | -3.1 | -2.8 | -3.5 |
| Telangana | -1.4 | -2.0 | -0.5 | -0.9 | -0.4 | -1.1 |

| **Supplementary Table S7.** Change in life disparity at birth ($e_{0}^{\dagger})$ estimates for Person, male and Female in India and states NFHS-4 (2015-16) to NFHS-5 (2019-21) and SRS (2015) to SRS (2020). | | | | | | |
| --- | --- | --- | --- | --- | --- | --- |
| **Region/State** | **changes in** $e_{0}^{\dagger}$**, NFHS 2015-16 to 2019-21** | | | **changes in** $e_{0}^{\dagger}$**, SRS 2015 to 2020** | | |
|  | **Person** | **Male** | **Female** | **Person** | **Male** | **Female** |
| **India** | -0.12 | -0.31 | 0.13 | -0.01 | 0.10 | -0.05 |
| **North** |  |  |  |  |  |  |
| Chandigarh | 2.06 | 0.01 | 4.51 | NA | NA | NA |
| Delhi | -2.50 | -3.65 | 1.20 | -2.11 | -5.09 | -0.18 |
| Haryana | -1.66 | -2.01 | -0.99 | 0.97 | 1.77 | 0.69 |
| Himachal Pradesh | 0.15 | 0.32 | -0.23 | 1.25 | -0.36 | 2.30 |
| Jammu Kashmir | -0.63 | 1.07 | -3.34 | -2.07 | -4.50 | -1.12 |
| Punjab | -3.09 | -3.69 | -2.39 | 1.23 | 1.33 | 1.16 |
| Rajasthan | -0.33 | 0.13 | -0.77 | 0.63 | 0.70 | 0.66 |
| Uttarakhand | -0.27 | -2.24 | 0.94 | 0.11 | -0.06 | 0.27 |
| **Central** |  |  |  |  |  |  |
| Chhattisgarh | -2.55 | -2.76 | -2.30 | -0.15 | -0.60 | 0.01 |
| Madhya Pradesh | 0.35 | -0.61 | 1.77 | 0.32 | 0.49 | 0.18 |
| Uttar Pradesh | -0.28 | -0.13 | -0.43 | 0.09 | 0.06 | 0.16 |
| **East** |  |  |  |  |  |  |
| Bihar | 0.32 | -0.18 | 0.77 | 1.33 | 0.79 | 1.87 |
| Jharkhand | 0.36 | -0.89 | 1.64 | -0.91 | -1.20 | -0.38 |
| Odisha | 0.91 | 0.89 | 1.10 | -0.38 | -0.19 | -0.57 |
| West Bengal | -0.83 | -1.00 | -0.54 | 0.58 | 0.93 | 0.39 |
| **Northeast** |  |  |  |  |  |  |
| Arunachal Pradesh | 1.80 | 0.80 | 3.24 | NA | NA | NA |
| Assam | 1.06 | -0.37 | 2.46 | -0.75 | -2.12 | 0.38 |
| Manipur | 0.63 | 0.46 | 0.70 | NA | NA | NA |
| Meghalaya | -5.06 | -4.44 | -5.84 | NA | NA | NA |
| Mizoram | 1.72 | -1.90 | 7.77 | NA | NA | NA |
| Nagaland | -1.46 | -0.33 | -4.36 | NA | NA | NA |
| Sikkim | -2.67 | 3.32 | -8.69 | NA | NA | NA |
| Tripura | -4.70 | -4.90 | -4.52 | NA | NA | NA |
| **West** |  |  |  |  |  |  |
| Dadara and Nagar Haveli | -10.15 | -23.82 | NA | NA | NA | NA |
| Daman and Diu | -5.71 | -19.29 | -3.23 | NA | NA | NA |
| Goa | 1.57 | -0.33 | 4.08 | NA | NA | NA |
| Gujarat | -0.49 | -0.86 | 0.01 | 0.55 | 0.88 | 0.44 |
| Maharashtra | -1.69 | -2.17 | -1.10 | 0.43 | 0.05 | 0.87 |
| **South** |  |  |  |  |  |  |
| Andaman and nicobar islands | -8.72 | -2.92 | -36.77 | NA | NA | NA |
| Andhra Pradesh | 2.87 | 3.26 | 2.44 | -0.13 | 0.56 | -0.85 |
| Karnataka | 1.67 | 1.06 | 2.15 | -0.63 | -0.21 | -0.70 |
| Kerala | -2.66 | -1.21 | -3.71 | -0.19 | 0.93 | -1.04 |
| Lakshadweep | -3.17 | -1.84 | -4.19 | NA | NA | NA |
| Puducherry | -3.38 | -7.62 | 3.10 | NA | NA | NA |
| Tamil Nadu | 1.84 | 2.26 | 1.53 | -2.05 | -0.89 | -2.89 |
| Telangana | 2.12 | 2.37 | 1.79 | -0.90 | 1.06 | -2.19 |
